# Estrogen improves the proliferation and differentiation of hBMSCs derived from postmenopausal osteoporosis through notch signaling pathway

**DOI:** 10.1007/s11010-014-2021-7

**Published:** 2014-04-22

**Authors:** Jin-Zhu Fan, Liu Yang, Guo-Lin Meng, Yan-shui Lin, Bo-Yuan Wei, Jing Fan, Hui-Min Hu, Yan-Wu Liu, Shi Chen, Jin-Kang Zhang, Qi-Zhen He, Zhuo-Jing Luo, Jian Liu

**Affiliations:** 1 Institute of Orthopedic Surgery, Xijing Hospital, Fourth Military Medical University, Xi′an, 710032 People’s Republic of China; 2 Department of Orthopaedics, First Affiliated Hospital, Chengdu Medical College, No. 278, Baoguang Road, Chengdu, SiChuan Province 610500 People’s Republic of China; 3 Department of Orthopaedics, Xi-jing Hospital, Fourth Military Medical University, No. 15, Changle West Road, Xi’an, Shaanxi Province 710032 People’s Republic of China

**Keywords:** Estrogen, Postmenopausal osteoporosis, HBMSCs, Notch signaling pathway, Cell proliferation, Cell differentiation

## Abstract

Estrogen deficiency is the main reason of bone loss, leading to postmenopausal osteoporosis, and estrogen replacement therapy (ERT) has been demonstrated to protect bone loss efficiently. Notch signaling controls proliferation and differentiation of bone marrow-derived mesenchymal stem cells (BMSCs). Moreover, imperfect estrogen-responsive elements (EREs) were found in the 5′-untranslated region of *Notch1* and *Jagged1*. Thus, we examined the molecular and biological links between estrogen and the Notch signaling in postmenopausal osteoporosis in vitro. hBMSCs were obtained from healthy women and patients with postmenopausal osteoporosis. Notch signaling molecules were quantified using real-time polymerase chain reaction (real-time PCR) and Western Blot. Luciferase reporter constructs with putative EREs were transfected into hBMSCs and analyzed. hBMSCs were transduced with lentiviral vectors containing human *Notch1 intracellular domain* (*NICD1*). We also used *N*-[*N*-(3, 5-diflurophenylacetate)-l-alanyl]-(S)-phenylglycine *t*-butyl ester, a γ-secretase inhibitor, to suppress the Notch signaling. We found that estrogen enhanced the Notch signaling in hBMSCs by promoting the expression of *Jagged1*. hBMSCs cultured with estrogen resulted in the up-regulation of Notch signaling and increased proliferation and differentiation. Enhanced Notch signaling could enhance the proliferation and differentiation of hBMSCs from patients with postmenopausal osteoporosis (OP-hBMSCs). Our results demonstrated that estrogen preserved bone mass partly by activating the Notch signaling. Because long-term ERT has been associated with several side effects, the Notch signaling could be a potential target for treating postmenopausal osteoporosis.

**Electronic supplementary material:**

The online version of this article (doi:10.1007/s11010-014-2021-7) contains supplementary material, which is available to authorized users.

## Introduction

Osteoporosis is a systematic skeletal disorder characterized by decreasing bone mineral density (BMD) and deteriorating bone structure due to imbalanced bone remodeling [1]. Fractures and secondly mortalities caused by osteoporosis have caused great harm and resulted in much cost to families and society. However, the essential underlying mechanism leading to osteoporosis is not fully understood, and how to delay the occurrence and progression of this disease remains a challenge in the clinic. In postmenopausal women, estrogen deficiency is closely associated with increased osteoporosis incidence and severity, and clinic data have demonstrated that postmenopausal women taking estrogen replacement therapy (ERT) had a reduced risk of pathological fracture compared with those not taking ERT. Estrogen improved the osteoblastic differentiation of hBMSCs through ER-α [2] or activating Wnt/β-Catenin signaling [3]. However, whether estrogen regulates osteoblastic differentiation through other signaling pathways, e.g., Notch signaling, is unclear.

The Notch signaling pathway is highly conserved and associated with cell-fate determination, self-renewal potential, and apoptosis [4]. Notch signaling pathway was found to be involved in the differentiation of BMSCs. The study performed by Ugarte [5] suggested that Notch signaling enhanced osteoblast differentiation and inhibited adipocyte differentiation of hBMSCs. Even more, Notch signaling was found to be involved in the differentiation of BMSCs into other cell types. When the expression of *Notch1* was blocked by mNotch-1 shRNA, the expressive level of several neuron-specific markers was increased, suggestive of an essential role of Notch signaling in the differentiation of BMSCs into neurons in vitro [6]. Other study [7] also showed that Notch signaling pathway mediated the process of BMSCs differentiation into endothelial cells. Notch receptors and their ligands (Jagged and Delta) are families of transmembrane proteins with large extracellular domains. Notch receptors become activated when bound with ligand, leading to the γ-secretase-dependent cleavage of the Notch intracellular domain (NICD). The NICD then translocates into the nucleus, where it interacts with the CSL family of transcriptional regulators and forms part of a Notch target gene-activating complex. Much attention has been focused on the crosslink between estrogen and Notch signaling in human cancers and brain development. Estrogen promoted the angiogenic process by activating Notch signaling in breast cancer [8], and it regulated brain development by blocking Notch signaling [9]. Whether Notch signaling also mediates the regulatory role of estrogen in hBMSCs has been not addressed. We analyzed the expression of key Notch signaling molecules between OP-hBMSCs and hBMSCs, then found that the expression of key Notch signaling pathway molecules was inhibited in the OP-hBMSCs. Whether Notch signaling mediates the regulation of estrogen on the proliferation and osteoblastic differentiation of hBMSCs and plays an essential role in the pathogenesis of osteoporosis remains unclear.

The aim of this study was to determine the molecular and biological links between estrogen and Notch signaling in the proliferation and differentiation of hBMSCs. In addition, we also investigated whether activation of the Notch signaling pathway could improve the proliferation and differentiation of OP-hBMSCs.

## Materials and methods

### Cell culture

Primary hBMSCs were obtained from bone marrow aspirates of 4 healthy women (*Control* 30.75 ± 2.22) and four patients with postmenopausal osteoporosis (*OP* 71.5 ± 2.38) after informed consent to the research protocol. Detailed information regarding the hBMSC donors is provided in Supplementary Table 1. Ethical approval was obtained from the ethics committee of the Fourth Military Medical University for this procedure (20110405-5). hBMSCs were harvested in a sterile environment and cultured as described previously [1].

### Luciferase reporter assay

When hBMSCs (4 × 10^5^ cells) harvested from healthy subjects grew to 80 % confluency in 24-well plates, they were transfected with a luciferase reporter plasmid containing different ERE fragments including *Notch1*-a (−2925 to 2913 bp region relative to the transcription start site, TSS), *Notch1*-*b* (−339 to 327 bp), *Jagged1*-*a* (−1339 to 1327 bp), *Jagged1*-*b* (−1627 to 1615 bp), *Jagged1*-*c* (−50 to 38 bp) EREs, or an empty vector with lipofectamine 2000. After 24 h, 10^−7^ M 17β-estradiol was added to the growth medium. After a 24 h incubation with 17β-estradiol, luciferase values were detected using the Dual-Glo^®^ Luciferase Assay System (Promega, USA). All procedures were performed according the instructions of the manufacturer.

### Osteoblastic induction

hBMSCs were seeded at 10^6^ cells/well on 6-well plates [2]. Upon reaching 70 % confluence, which counted as day 0, the cells were exposed to osteoblastic differentiation medium supplemented with either 10^−7^ M 17β-estradiol or 10^−5^ M DAPT. The osteoblastic induction was carried out as described previously [10]. ALP and Alizarin red staining were performed to assess the mineralization ability as previously reported on days 7 and 14 [11]. Each experiment was performed in triplicate and repeated three times.

### MTT assay

hBMSCs of different groups in the exponential growth phase were plated at 2 × 10^3^ cells/well in 96-well plates. MTT assay were carried out as described previously [10].

### Colony formation assay

Briefly, cells were plated in 6-well plates at 100 cells/well. Whole experiment was repeated three times. Colony formation assay were carried out as described previously [10].

### Quantitative real-time PCR

The mRNA levels for *Notch1*, *Jagged1*, *Hes1*, *Runx2*, *ALP,* and *Osterix* were measured by quantitative real-time PCR as described previously [12]. Target genes expression was normalized to the reference gene GAPDH. The 2^−ΔΔCt^ method was used to calculate relative gene expression. The PCR products were subjected to melting curve analysis and a standard curve to confirm correct amplification. All the real-time PCRs were performed in triplicate.

### Western blotting

The expression levels of JAGGED1 and NOTCH1 protein from cell samples were analyzed as described previously [13]. All the antibodies used for western blotting were from CST, Boston, USA.

### Generation of lentivirus vector constructs and transduction of primary hBMSCs

Lentiviral transfer vectors were created with the human *NICD1* ORF (+921 ~ +2409 AA). Transgenes were amplified from a human complementary DNA library (MegaMan; Stratagene, La Jolla, CA, USA) and directionally inserted into the GV205 vector, which was purchased from GeneChem, Shanghai. An Ubi-MCS-3FLAG internal site fragment was added to check the transfection efficiency. An empty vector was used as a negative control. Virus vector particles were obtained by transiently transfecting 293 T cells with transfer vector and packaging plasmids as previously described [14–16]. Primary hBMSCs were transduced once they had reached confluency with 1:10 diluted neat virus vector supernatant at 37 °C for 12 h. The transduction efficiency was quantified by real-time PCR and Western blotting.

### Statistical analysis

All experiments were performed repeatedly in three times. The data are expressed as the mean ± SD, and samples were evaluated by the ANOVA test using SPSS 16.0.

## Results

### 17β-estradiol enhanced osteoblastic differentiation and activated Notch signaling in hBMSCs in a ligand-dependent manner

In our previous studies, the expression level of key Notch signaling molecules was compared in hBMSCs between patients with postmenopausal osteoporosis and healthy women by real-time PCR. Our results showed that Notch signaling was impaired in OP-hBMSCs (Supplementary Fig. 1).

To test the relationship between estrogen and osteoblastic differentiation of OP-hBMSCs, we exposured OP-hBMSCs to an osteogenic induction medium containing 17β-estradiol and detected the expression of the osteoblastic markers *Runx2*, *ALP*, and *Osterix* by real-time PCR (Supplementary Fig. 1). On days 3 after osteogenic induction, the 17β-estradiol-treated OP-hBMSCs significantly expressed an elevated level of *Runx2* and Osterix. Furthermore, the addition of ICI-182780 (ICI; an ER antagonist) almost completely blocked the enhancement in osteoblastic differentiation that was induced by 17β-estradiol (Supplementary Fig. 2). Therefore, our findings demonstrated that 17β-estradiol enhanced osteoblastic differentiation of OP-hBMSCs.

**Fig. 1:**
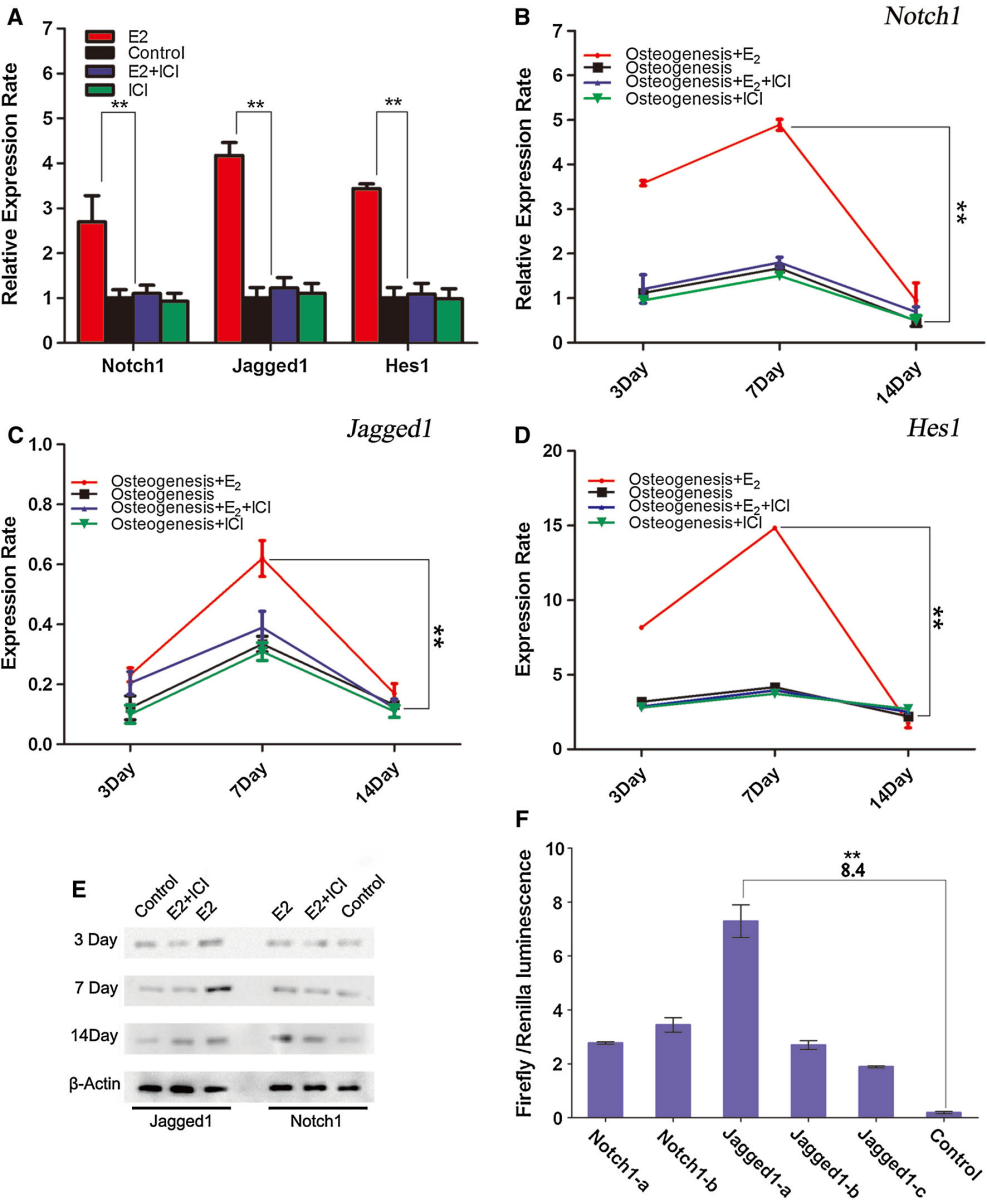
17β-estradiol (E_2_) activated Notch signaling in hBMSCs in a ligand-dependent manner. Expression level of key Notch signaling molecules was analyzed in hBMSCs from postmenopausal osteoporosis patients after 17β-Estradiol and ICI Treatment (**a**). *Notch1, Jagged1*, and *Hes1* genes were evaluated in a time course assay by real-time PCR (**b**–**d**) and Western blotting (**e**) in the progress of osteoblastic differentiation. Role of 17β-estradiol on activation of imperfect EREs in the 5′-Flanking region of *Notch1* and of *Jagged1* was assessed by luciferase assay. ** *p* *<* 0.01, compared to the control (**f**). All results were representative of three independent experiments. Results are statistically valid

To further understand the relationship between estrogen and Notch signaling, we first tested whether estrogen treatment could reverse the defective Notch signaling in OP-hBMSCs. When OP-hBMSCs were treated with 17β-estradiol, the expressions of *Notch1* and *Jagged1* were found to be significantly up-regulated. *Hes1*, which is known as a target of canonical Notch signaling, also showed more than threefold increase of its expression level. Furthermore, the addition of ICI-182780 almost completely blocked the increase in Notch signaling that was induced by 17β-estradiol (Fig. 1a).

We further tested the relationship between estrogen and Notch signaling during the osteoblastic differentiation of hBMSCs. Real-time PCR and Western blotting were performed to evaluate the expression of *Notch1*, *Jagged1*, and *Hes1* on days 3, 7, and 14 of osteoblastic differentiation. 17β-estradiol was found to significantly increase the expression of these key Notch signaling molecules at the onset of differentiation but reduced them on osteoblastic differentiation days 14 when most of hBMSCs had differentiated into mature osteoblasts [12]. The addition of ICI into the culture medium blocked this 17β-estradiol effect (Fig. 1b–d). Compared to the mRNA level, the protein levels of NOTCH1 and JAGGED1 were slightly increased after 17β-estradiol treatment. On days 7, JAGGED1 was significantly increased, indicating that 17β-estradiol might enhance Notch signaling by promoting JAGGED1 expression during osteoblastic differentiation (Fig. 1e). This finding indicated that Notch signaling might mediate estrogen’s regulation on hBMSCs. However, the molecular link between estrogen and Notch signaling in hBMSCs was unclear.

To determine the potential molecular link between Notch signaling and estrogen, we examined the binding affinity of estradiol to several EREs in the 5′-flanking region of the *Jagged1* and *Notch1* genes. Using the luciferase reporter system, the luciferase activity of hBMSCs, which were transfected with plasmids containing different EREs and the luciferase gene, was examined after supplying 17β-estradiol. After 17β-estradiol treatment for 24 h, the luciferase activities of all groups of hBMSCs transfected with different ERE plasmids were increased compared to that of hBMSCs transfected with a control plasmid (Fig. 1f). The luciferase activity was nearly eightfold increased for the Jagged1-a ERE fragment (*p* < *0.01*), which is much greater than that of the other ERE fragments. This result indicated that 17β-estradiol activated Notch signaling mainly through the activation of the Jagged1-a ERE. Together, these findings demonstrated that 17β-estradiol promotes ligand-induced Notch signaling in hBMSCs.

### 17β-estradiol promoted hBMSC proliferation and differentiation partially by activating the Notch signaling pathway

**Fig. 2:**
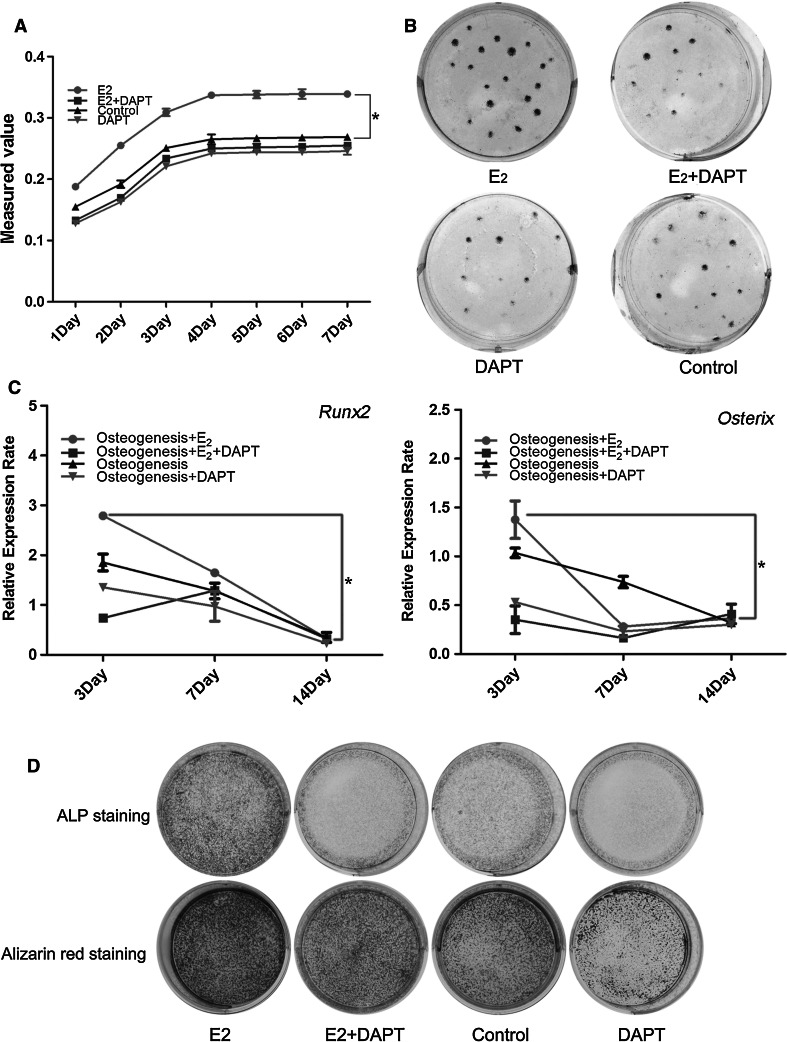
17β-estradiol promoted hBMSCs proliferation and differentiation partially by activating Notch signaling pathway. Growth curves (**a**) and plate colony formation assay (**b**) of hBMSCs cultured in different conditions.* *p* < *0.05*, compared to the control. Osteogenic markers' expression with or without the addition of DAPT by real-time PCR. * *p* < *0.05*, compared to the control (**c**). ALP Staining and Alizarin red staining with or without the addition of DAPT (**d**)

To elucidate whether 17β-estradiol could promote the proliferation and osteoblastic differentiation of hBMSCs partially by activating the Notch signaling pathway, we used DAPT, a Notch signaling antagonist, to block Notch signaling in hBMSCs. First, we found that 17β-estradiol significantly increased the number of hBMSCs, while DAPT addition reversed the elevated number of hBMSCs (Fig. 2a). The colony formation assay demonstrated that blocking Notch signaling decreased the proliferative ability of estrogen-induced hBMSCs in vitro (Fig. 2b).

Cultured within osteoblastic differentiation medium, hBMSCs underwent sequential osteoblastic differentiation. We evaluated the osteoblastic differentiation of hBMSCs by examining the expression of the osteoblastic markers *Runx2* and *Osterix* by real-time PCR (Fig. 2c). On days 3 after osteoblastic induction, 17β-estradiol stimulated hBMSCs to express elevated levels of *Runx2* and *Osterix*, while DAPT addition reversed the 17β-estradiol effect. ALP and Alizarin red staining on days 7 and 14 also demonstrated the increased differentiation ability, which was induced by 17β-estradiol and blocked by DAPT (Fig. 2d).

Our current findings demonstrated that 17β-estradiol stimulated the proliferation and differentiation of hBMSCs, while DAPT blocked the effect of 17β-estradiol. Together with our finding that Notch signaling pathway was impaired in OP-hBMSCs and 17β-estradiol enhanced its activity in a ligand-dependent manner, we believed that 17β-estradiol regulated the proliferation and differentiation of hBMSCs by activating the Notch signaling pathway.

### The Notch signaling pathway enhanced the proliferation and differentiation of OP-hBMSCs

**Fig. 3:**
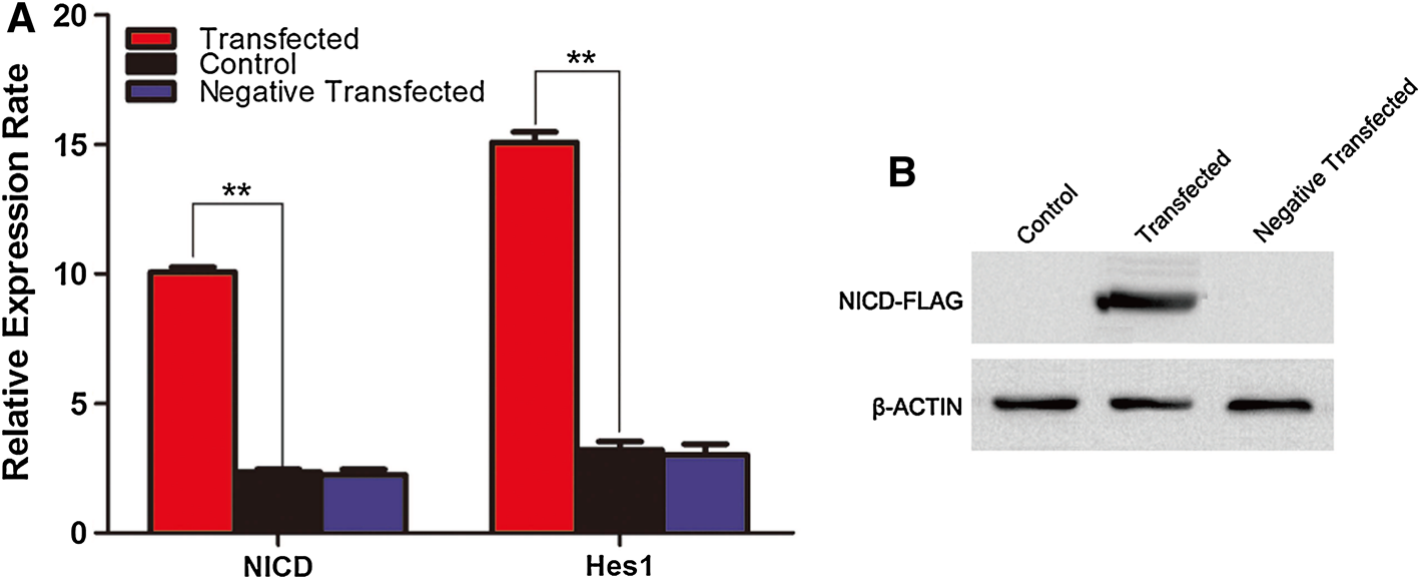
Overexpression of *notch1 intracellular domain* (*NICD1*) in hBMSCs from postmenopausal osteoporosis patients. Real-time PCR results of *NICD1* and Notch target gene *Hes1* demonstrate up-regulated expression in *NICD1* transgene expressing cells compared to the negative control-transduced cells. ** *p* < *0.01*, compared to the control (**a**). hBMSCs were transduced successfully confirmed by Western blotting (**b**)

To further address whether Notch signaling mediates the enhanced proliferation and osteoblastic differentiation of hBMSCs by estradiol, we used a lentiviral vector system to efficiently overexpress *Notch*-*1 intracellular domain* (*NICD1*) in OP-hBMSCs. The lentiviral transduction of OP-hBMSCs was confirmed by detecting *NICD1* expression by real-time PCR and FLAG expression by Western Blotting (Fig. 3a, b). In addition, Notch signaling activation was determined by quantifying the expression of *Hes1* using real-time PCR (Fig. 3a). By enforced *NICD1* overexpression, the *Hes1* expression level nearly increased fivefold (*p* < *0.01*). An empty lentiviral vector was used as a negative control.

**Fig. 4:**
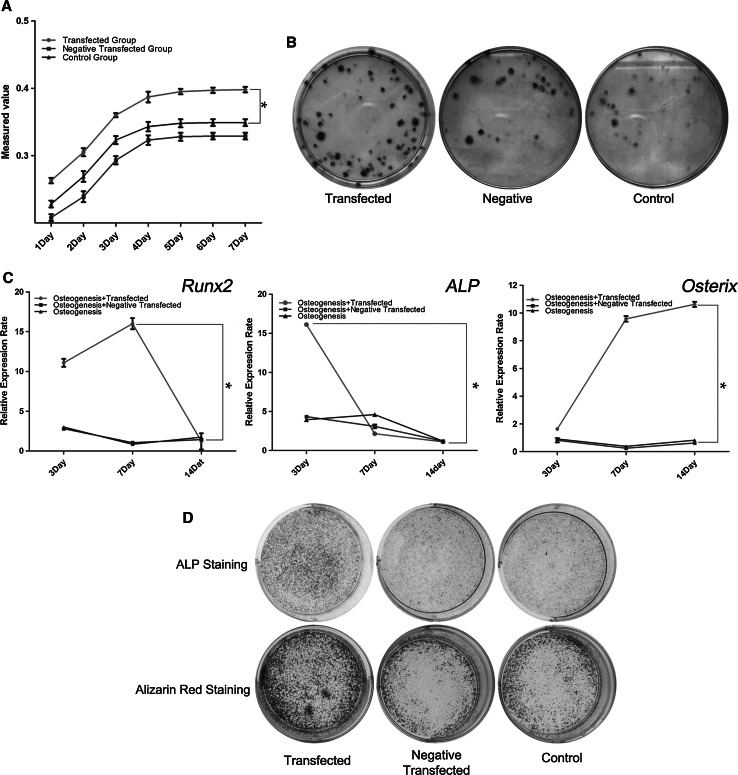
The Notch signaling pathway enhanced the proliferation and differentiation of OP-hBMSCs. Growth curves (**a**) and plate colony formation assay (**b**) of OP-hBMSCs illustrate improved vitality in response to Notch activation.* *p* < *0.05*, compared to the control. A significant increase in *Runx2*, *ALP*, and *Osterix* is observed in *NICD1* transgene positive OP-hBMSCs. * *p* < *0.05*, compared to the control (**c**). ALP staining and Alizarin *red* staining demonstrated the elevated osteogenic differentiation and mineralization in response to Notch signaling (**d**)

*NICD1*-transduced OP-hBMSCs appeared as typical clusters of spindle-shaped cells, compared with the fibroblast-like OP-hBMSCs in the control group. To elucidate whether Notch signaling could rescue the defective proliferation of OP-hBMSCs, the cell proliferative rate was evaluated by a growth curve and colony forming assay. (*p* < *0.05*, Fig. 4a, b). These data demonstrated that the enforced expression of *NICD1* efficiently rescued the proliferative ability of OP-hBMSCs.

Within osteogenic induction medium, the osteoblastic differentiation of OP-hBMSCs was analyzed by detecting the expression of the osteoblastic markers *Runx2*, *ALP*, and *Osterix* (Fig. 4c). On days 3 after osteogenic induction, the *NICD1*-transduced OP-hBMSCs significantly expressed an elevated level of *Runx2*, *ALP*, and *Osterix*. Along with an induction time course, the increasing levels of *Runx2* and *ALP* gradually declined and the expression of *Osterix* continuously increased. ALP and Alizarin red staining of the OP-hBMSCs on days 7 and 14 also demonstrated the elevated differentiation ability of *NICD1*-transduced OP-hBMSCs (Fig. 4d). Together, our findings demonstrated that the defective proliferation and differentiation of OP-hBMSCs could be rescued by up-regulating Notch signaling in vitro.

## Discussion

Previously, some groups have reported that the decline in the number and defective osteoblastic differentiation capacity of hBMSCs were the important factors contributing to osteoporosis [17]. Thus, modulation of the proliferation and differentiation of hBMSCs was believed to be a new strategy for treating osteoporosis. Our study demonstrated for the first time that 17β-estradiol promoted the proliferation and differentiation of hBMSCs partially by activating the Notch signaling pathway.

The molecular link between estrogen and Notch signaling has been detected in breast cancer and endothelial cells [9, 13] but not in hBMSCs. Those studies demonstrated that the estrogenic compound genistein could down-regulate Notch1 in prostate cancer cells. In contrast, estrogen was also shown to increase the number of tumor microvessels through the activation of Notch signaling. In turn, Notch1 could activate ERα-dependent transcription in these cells in the presence or absence of estradiol [18]. In our previous study, we noted that Notch signaling was impaired in hBMSCs from postmenopausal osteoporosis patients. Our study further found that 17β-estradiol could activate *Jagged1* gene transcription mainly through Jagged1-a (−1339 to 1327 bp). This finding stated that *Jagged1* expression was enhanced by 17β-estradiol, and estrogen enhanced Notch downstream signaling in a ligand-dependent manner. The results are consistent with a study by Soares [13], which was performed in MCF7 cells. However, in MCF7 cells, estrogen regulated Notch signaling through Jagged1-b and not through the Jagged1-a ERE, which was demonstrated in our study. The difference in results may be due to the different cells used in studies.

It is well known that *Runx2* and *Osterix* have crucial roles in osteogenesis [19, 20]. Qi Shen et al. [21] also demonstrated that *Hes1* cooperated with *Runx2* to stimulate the *Osteopontin* or *Osteocalcin* promoters and then enhance osteogenesis. *Osterix* is downstream of *Runx2* and has been shown to have a major role in the later stage of osteogenesis [20]. Here, the overexpression of NICD1 significantly increased the levels of *Runx2* and *Osterix* of *NICD*-transduced OP-hBMSCs, evaluated by real-time PCR, indicating the activation of Notch signaling improved the proliferation and enhanced osteoblastic differentiation of OP-hBMSCs.

Notch signaling regulates osteoblastic differentiation in a cell type- and cell stage-dependent manner [22, 23]. Previously, transient Notch activation had been suggested to stimulate the osteoblastic differentiation of MC3T3-E1. However, some studies suggested that Notch signaling impaired the osteoblastic differentiation of the Kusa, MC3T3, and ST-2 cell lines [22, 24]. Matthew J. Hilton demonstrated the opposite effects of Notch signaling on osteogenesis during different stages of mouse development [25]. When these precursors began to undergo osteoblast differentiation, the activation of Notch signaling could promote them to osteoblast pool; however, when these cell already finished the early differentiation, Notch signaling might be not necessary for them to continue their late differentiation to osteocyte. Our results in this study also showed similar finding, and Notch signaling also promoted the proliferation and early osteoblast differentiation of hBMSCs derived from healthy women and patients with postmenopausal osteoporosis. Of course, our study did not exclude the possible regulation of Notch signaling by other factors, e.g., aging.

Based on our data, we demonstrated that estrogen regulated the differentiation and proliferation of hBMSCs partially by activating the Notch signaling pathway. An optimal utilization of Notch signaling could effectively improve bone mass of postmenopausal osteoporosis. The Notch signaling pathway could be a potential strategy for treating postmenopausal osteoporosis in vivo. However, Notch signaling also contributes to cancer and tumor angiogenesis. Therefore, balancing the efficacy to treat postmenopausal osteoporosis and Notch signaling side effects must be considered for future clinical applications. In the future, we hope to develop a Notch activator only targeting and promoting osteogenic differentiation, and to maintain bone mass. Meanwhile, it could be applied to some fragile bones which are easier to fracture, so as to prevent fractures and improve the quality of life of postmenopausal women.

## Electronic supplementary material

Below is the link to the electronic supplementary material.

Supplementary material 1 (DOCX 1041 kb)
