# Supplementary material for: Estrogen improves the proliferation and differentiation of hBMSCs derived from postmenopausal osteoporosis through notch signaling pathway
Source: Mol Cell Biochem. 2014 Apr 22;392(1):85–93. doi: 10.1007/s11010-014-2021-7 (PMC4053611; doi:10.1007/s11010-014-2021-7)
Supplement: Supplementary file 1 — Supplementary material 1 (DOCX 1041 kb) [file 11010_2014_2021_MOESM1_ESM.docx]

**Supplementary materials and data:**

1. We collected and cultured primary hBMSCs from bone marrow aspirates of 4 healthy women (30.75±2.22) and 4 patients with postmenopausal osteoporosis (71.5±2.38). Detailed information of hBMSC donors is provided in **Supplementary Table 1**. Ethical approval was obtained from the ethics committee of the Fourth Military Medical University for this procedure (20110405-5).

| **Supplementary Table 1.** Characteristics of hBMSC donors | | | |
| --- | --- | --- | --- |
| Donor | Age | Sex | T-score/BMD |
| C1 | 32 | F | -0.3 |
| C2 | 28 | F | -0.5 |
| C3 | 30 | F | -0.5 |
| C4 | 33 | F | -0.9 |
| OP1 | 69 | F | -2.8 |
| OP2 | 70 | F | -3.2 |
| OP3 | 74 | F | -3 |
| OP4 | 73 | F | -2.7 |

hBMSC: human bone marrow stromal cell, M: male, F: female

Total RNA was extracted from cells of the 3^rd^ passage and *Notch1, Jagged1* and *Hes1* were detected by real-time PCR. And statistic analysis based on current number of patients and controls showed the expression of *Notch1, Jagged1* and *Hes1* were significantly decreased in OP-hBMSCs (Supplementary Fig.1).


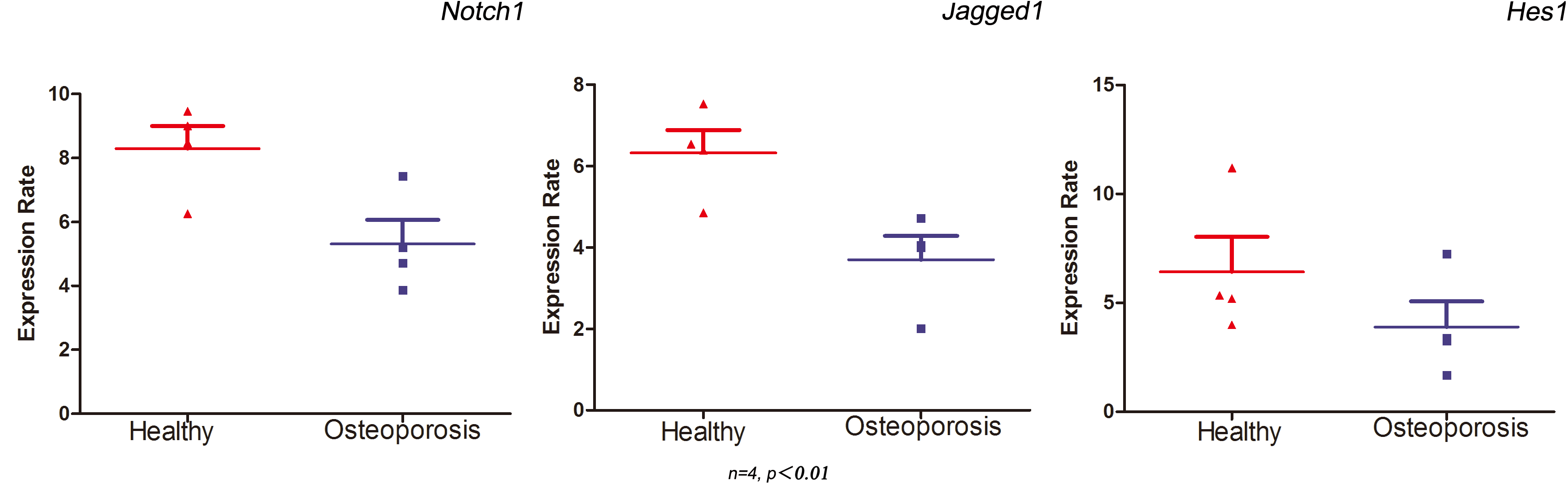


**Supplementary Figure 1:** The expression of *Notch1, Jagged1* and *Hes1* were significantly decreased in OP-hBMSCs (p<0.01).

2. Total RNA was extracted from cells of the 3rd passage and OB marker genes were detected by real-time PCR. Because these data have been reported in 2006 by Hong L et al (*Hong L, Colpan A, Peptan IA (2006)* *Modulations of 17-beta estradiol on osteogenic and adipogenic differentiations of human mesenchymal stem cells. Tissue Eng 12 (10):2747-2753. doi:10.1089/ten.2006.12.2747*). This is also the reason why we did not put this data in our manuscript.

Now we add this result into **Supplementary data** for your consideration. And statistic analysis showed the expression of OB marker genes were significantly promoted by 17β-estradiol in OP-hBMSCs (Supplementary Fig.2).


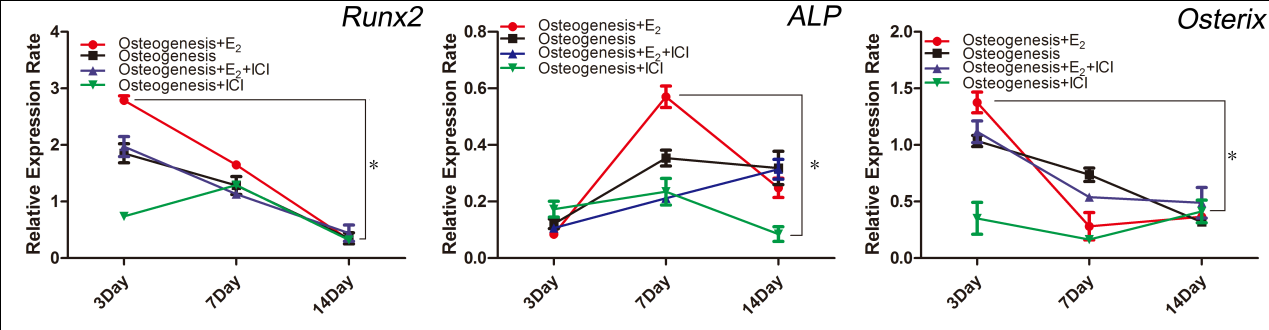


**Sup. Fig.1:** The expression of OB marker genes were significantly promoted by 17β-estradiol in OP-hBMSCs (**P<0.05*).

3、


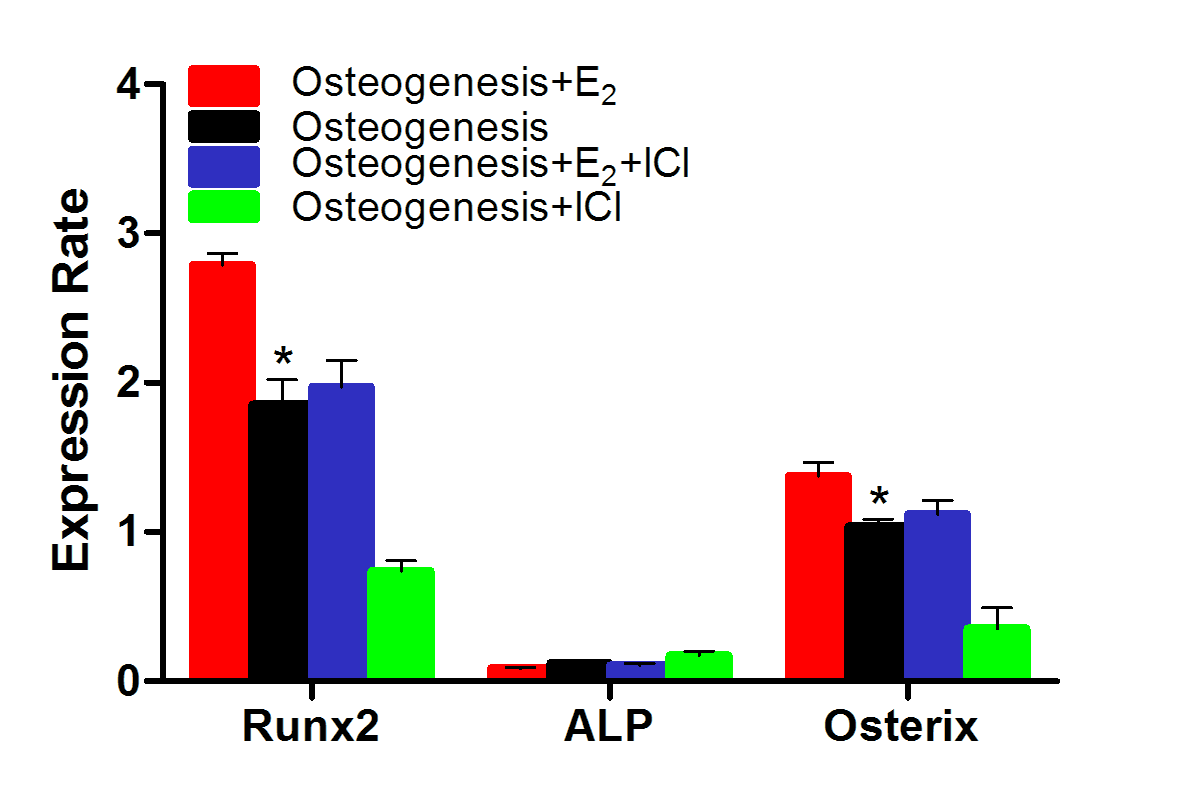


**Sup. Fig.2:** The expression of OB marker genes were significantly promoted by 17β-estradiol in OP-hBMSCs on days 3 after osteogenic induction (**P<0.05*).
